# Effects of* Matricaria Recutita* (L.) in the Treatment of Oral Mucositis

**DOI:** 10.1155/2018/4392184

**Published:** 2018-06-12

**Authors:** Vânia Thais Silva Gomes, Raimundo Nonato Silva Gomes, Maria Silva Gomes, Walderez Moreira Joaquim, Eliana Campêlo Lago, Renata Amadei Nicolau

**Affiliations:** Research and Development Institute, University of Vale do Paraiba, São José dos Campos, SP, Brazil

## Abstract

**Objective:**

The objective of this study was to review the effects of the* Matricaria recutita* (L.) in the treatment of oral mucositis.

**Methodology:**

The online search was performed in the period from June 2016 to April 2018 by means of databases LILACS (Latin American and Caribbean Center on Health Sciences Information), SciELO (Scientific Electronic Library Online), and MEDLINE (Medical Literature Analysis and Retrieval System Online). The consultation was restricted to the years 1991 to 2018 with the aim of elucidating the effects of* Matricaria recutita* in the treatment of oral mucositis.

**Results:**

The final sample consisted of 21 studies, of which 10 were developed in animals and 11 in humans, published from 1991 to 2017, with a total sample of 644 patients. The total number of patients treated with* Matricaria* included in 11 studies was 364, while in the control groups the total number was 280. In experimental studies, animal models used were rats and the sample size ranged between 36 and 105 animals submitted to the induction of oral mucositis, where 4 studies used an intraperitoneal injection of 5-fluorouracil, while 7 induced lesion in the mucosa. From the data collected, it should be noted that both studies with humans and with animals showed significant effects. In this way, there is strong evidence for the discussion on the therapy; however, it should be noted that more studies are developed in order to clarify the most appropriate protocol for the prevention and treatment of injuries.

**Conclusion:**

According to the results found in this study,* Matricaria recutita* appeared to be a promising alternative for the treatment of oral mucositis. However, due to the great variability in the various types of intervention, more controlled double-blind randomized clinical studies are necessary to ensure the best protocol for treating oral mucositis.

## 1. Introduction

The oral mucositis is a common inflammation in patients with malignant neoplasms, undergoing antineoplastic therapy; its symptoms predispose the oncological patient to various serious complications. Its incidence is 75–100% among patients who perform hematopoietic stem cell transplantation; 40–85% of incidences occur in patients during chemotherapy and/or radiotherapy [1, 2].

The toxicity caused by antineoplastic agents generates an inflammatory response mediated by reactive oxygen species, proinflammatory cytokines, interleukin-1, interleukin-11, and interleukin-6, which harm not only the tissues but also adjacent cells, causing injury in mucosal cells and thus reducing the epithelial thickness, resulting in ulcers [3, 4].

Due to the inflammatory condition, the oncological patient can present difficulties in basic oral functions such as speech and chewing, with recurrent complications like dysgeusia, intense pain in swallowing, nutritional deficiencies, and risk of secondary infections. This whole range of events significantly interferes in the patient's quality of life. Furthermore, in the most severe degrees, oral mucositis may require partial or complete interruption of the antineoplastic treatment, thereby increasing the proliferation of tumor cells and hindering cancer control [5, 6].

Strategies to prevent and/or treat oral mucositis are still not well clarified because there is no defined protocol. However, some measures are employed to reduce its incidence and severity, such as basic oral care protocols, the 0.12% chlorhexidine digluconate, anti-inflammatory therapy, biological response modifiers, cryotherapy, low-intensity laser therapy, and the use of plant extracts as* Matricaria recutita* [7].

*Matricaria recutita *Linnaeus (Asteraceae) is known as chamomile and has been studied for years because of its agronomic and phytochemical aspects. It is a plant widely used in traditional medicine for its antioxidant, antimicrobial, and anti-inflammatory action. It features more than 200 constituents such as terpenoids, flavonoids, coumarins, fatty acids, alkaloids, polysaccharides, and glycoside derivatives. This plant has been used in the treatment of oral mucositis in order to provide relief and comfort to the painful symptoms of the patient [8, 9]. In the light of the considerations raised, this study aims to review the effects of* M. recutita* in the treatment of oral mucositis.

## 2. Methods

### 2.1. Search Strategy

The online search was conducted during the period from June 2016 to April 2018 through the databases LILACS (Latin American and Caribbean Center on Health Sciences Information), SciELO (Scientific Electronic Library Online), and MEDLINE (Medical Literature Analysis and Retrieval System Online). The query was restricted to the years of 1991 to 2018 in order to elucidate the effects of* Matricaria recutita* in the treatment of oral mucositis. The descriptors were selected through consultations in DeCS (Descriptors in Health Sciences) and MeSH (Medical Subject Headings): “*Matricaria recutita*,” “oral mucositis,” “stomatitis,” and “cancer.” The same descriptors were used in all the virtual libraries to standardize the research. Initially, two researchers sought articles that met the criteria of selection and examined the titles and abstracts of studies available in the databases searched. Then, the articles did not heed eligibility criteria or duplicate items were not included in this study. From this assessment, the researchers selected the studies that were available in full by the regular portal access of the Coordination for the Improvement of Higher Education Personnel (CAPES).

### 2.2. Selection Criteria of the Studies

This work included studies that addressed mucositis and stomatitis caused by chemotherapy and/or radiation, as well as studies that used* Matricaria* as therapeutic approach. Due to the scarcity of studies that used* Matricaria* in treatment of mucositis or stomatitis in humans, we decided to include studies on animals in order to get more results on the effects of the plant in the prevention and healing of injuries. We adopted the following inclusion criteria: type of study (clinical trials phases I, II, III, and IV, controlled clinical trials, randomized controlled clinical trials, and observational studies of the cohort), subject of research (human beings and animals ), language of publication (English and Spanish), and year of publication (1991 to 2018). There were deleted articles that did not heed eligibility criteria.

### 2.3. Data Collection and Quality of Studies

The relevant data for this study were obtained by two researchers through form developed from the identification of the most significant data for this research. We discussed information about author and year of publication. The data regarding the population of article covered data such as sample, type of cancer, neoplastic treatment type, and route of administration of the agent, as well as the type of treatment to the control group. The type of intervention was also included, but note that there is no standardization of the vehicle used, form of administration, and duration of treatment. Therefore, in order to evaluate the methodological quality of the studies, we used a scale of quality. The criteria for assessing the quality of scale through studies were randomization, double-blinding, and masking all the losses and exclusions. The scale used to evaluate the methodological quality of the articles proposes a maximum of five points: one point for each Yes, an additional point for the randomization, and other additional masking points (Table 1). Even if the expression double-blind is not mentioned in the text but there is the researcher's description and patients' masking, this item is included in the context. The study quality is obtained by its amount of points, considering it with two or less points as a study of poor quality [10].

## 3. Results

The online search resulted in 65 jobs for the descriptors used. 39 articles were selected for thorough evaluation of the inclusion and exclusion criteria, being considered potentially relevant studies. After analysis, it was found that five were review articles and four dealt with in vitro study. In addition, two studies were not* Matricaria* and two works were comments from articles and were excluded.

The final sample consisted of 21 studies, of which 10 were developed in animals and 11 in humans, published between 1991 and 2017, with a total sample of 644 patients. The total number of patients treated with* Matricaria* included in 11 studies was 364, while in control group the total number was 280. In the sample, the overall frequency of head and neck neoplasms was 28% and 36% were undergoing treatment for hematologic diseases, 18% did not declare the type of neoplasm, 9% had gastric cancer, and 9% were bearers of non-Hodgkin lymphoma (Table 2).

With regard to oncological treatment modality, three studies have treated their patients with radiotherapy, patients were treated with chemotherapy in six other articles, and in two other studies the patients were treated with hematopoietic cell transplantation. Nine studies used the grading scale for oral mucositis, whereas only one study used the NCI oral toxicity scale. One study used the visual analogue scale and compass.

In view of the scarcity of articles related to the theme of this review, 10 studies were added which addressed the anti-inflammatory effects and lesions healing induced in* Matricaria* animals. In experimental studies, animal models used were mice and the sample size ranged from 36 to 105 animals undergoing induction of oral mucositis, where 4 studies used intraperitoneal injection of 5-fluorouracil, while 7 studies induced injury in mucosa.

From the data collected, it is observed that both studies with humans and with animals showed significant effects. In this way, there is robust evidence for the discussion on the therapy; however, it should be noted that more studies are developed in order to clarify the most appropriate protocol for the prevention and treatment of injuries.

According to the studies presented in Tables 2 and 4, there are different ways of preparation and administration of* Matricaria recutita* and standardization in different types of intervention, with varied concentrations and different constituents that can lead to controversial results. As to the duration of treatment, there is no standardization in treatment time, which can range from 5 to 67 days; however, you can see positive results in the early days. There are no studies in the literature addressing the presence of adverse events in any of the interventions. Quality analysis was performed only in the studies developed in humans, in which only 10 articles were analyzed; one article was excluded from this analysis because it did not meet the scale criteria. The result of the quality evaluation revealed that 90% (9/10) of the articles analyzed were of good quality (Table 3).

## 4. Discussion

The vast majority of the synthetic agents used to prevent or treat oral lesions resulting from cancer treatment present side effects and can interact with other drugs. However, the natural medicines are presented as a promising alternative; however, few studies evaluate the effects of these agents in the reduction of injuries. This study sought to gather through a literature review articles that used* Matricaria* to treat oral lesions; this search resulted in 21 studies in humans and animals.

Most of the studies found showed consistent evidence and assessed the effects of* M. recutita *in the control of oral mucositis to be positive. As for the other outcomes, the few publications indicate that such effects are caused due to the inhibition of nitric oxide and nitric oxide synthase, blocking of the transcription factor NF-k*β*, and inhibition of COX-2 and metalloproteinase-9 which reduce discomfort and severity of oral mucositis.

*M. recutita* is a plant widely used in traditional medicine due to its therapeutic properties that come from its chemical constituents. Its antioxidant effects inhibit free radicals and reduce the levels of IL-1b and TNF-*α*, which causes a histopathological and clinical improvement. The results of the present study corroborate data obtained by [25] regarding the levels of mediators influenced by action of vegetable extract, which directly and indirectly affect inflammatory and repair phase in oral mucositis.

According to the results of this study, there is a great variability in the types of intervention regarding the vehicle, the used concentration, mode of application, and time of treatment. However, the use of ointment and antiseptic mouthwash prevailed; the average treatment time was 15 days, using the intervention twice a day on average. A comparative analysis of standards and the results obtained in these studies is necessary, so that they may be reproduced and used in clinical practice [30].

Among the selected studies, eight compared* M. recutita* with other forms of treatment. In a study that compared a mouthwash with 1% concentration of* Matricaria recutita* (group I) with a placebo mouthwash (group II), group I showed significant reduction in the 2^nd^, 3^rd^, and 4^th^ assessment, showing that the symptoms, healing period, and the number of lesions decrease after two days using the mouthwash, which corroborates the results of this review [31].

Oral mucositis in oncological patients is associated with the dose of the chemotherapy drugs and the type and scope of their administration time; consequently, these elements can vary depending on the used protocol, as well as the clinical characteristics of the patient [32].

Based on the results, it was found that the studies showed different results in the treatment of oral mucositis. This fact can be explained by the large heterogeneity of protocols, different prophylactic measures, and time duration of the intervention. For* Matricaria* commercialization as therapeutic agent of oral mucositis, the dosages need to be standardized in order to minimize any risk to the patient.

In an analysis of the effectiveness of a mouthwash containing 1% of* Matricaria recutita* extract in patients with gingivitis and fixed orthodontic appliances, the authors found that mouthwash had significantly reduced the rate of gingival bleeding after 15 days of treatment, compared with the placebo group. However, this reduction was similar to the group that used the 0.12% chlorhexidine and the difference between the groups was not significant [18].

One study evaluated the effect of* Matricaria recutita* and triamcinolone in patients with aphthous stomatitis and found that the ulcer size reduced on the 3^rd^ day as well as pain on the 3^rd^ and 6^th^ days in both groups. However, the triamcinolone was superior to* M. recutita* on the 6^th^ day [33]. This finding supports the results found in the study that showed that the extract provided pain relief in 82% of patients with stomatitis after 5, 10, and 15 minutes; the authors concluded that the analgesic effect improved the patients' quality of life [21].

In this article, we present the current clinical evidence on the effects of* Matricaria* for prevention and treatment of oral mucositis. The most common limitations observed in the studies were the sample size, the diversity of protocols, duration of intervention, and the different treatments used in the control group, making it difficult to have a definitive conclusion of the effectiveness of chamomile on mucositis.

The results found in this review demonstrate the therapeutic potential of* Matricaria recutita* in the treatment of oral mucositis. The different protocols used as well as the clinical characteristics of the patients in the selected studies indicate that there may be, or not, greater involvement of the oral mucositis, preventing, in this way, a more careful analysis and hindering its proper management. In this review, all studies showed significant reduction of oral mucositis after using* M. recutita*, with lower incidence, severity, and time of involvement, which indicates that this extract is effective in the studied involvement.

Thus, one can infer that* Matricaria recutita* can directly inhibit COX-2 and the synthesis of inflammatory mediators, such as prostaglandin E2 [34]. In this way,* M*.* recutita* appears to be an option for the treatment of oral mucositis, because it can inhibit the action of proinflammatory cytokines, influence the chemotaxis of leukocytes and COX-2 way and lipopolysaccharide, and activate macrophages. Furthermore, it can influence the repair of injuries, reduce neutrophil elastase and metalloproteinase-9 and inhibit the transcription factor Natural Killer Cell (NK); thus, it can reduce the discomfort and severity of oral mucositis during the oncotherapy [25, 26]. Therefore, for feedstock to be marketed as a therapeutic agent of oral mucositis, it is necessary to standardize dosages in order to minimize any risk to the patient.

Table 5 presents the main features of* Matricaria recutita* effects in animal and human models. The treatment of oral mucositis has been only palliative in order to minimize the pain and control the possible infections, contributing to the repair process. Thus, the plant extracts have shown promising results: low cost, no side effects [14, 27, 35], and easy self-application.

## 5. Conclusion

According to results, it is concluded that the studies that evaluate the effects of* Matricaria* on oral mucositis are scarce. However, this proved to be a promising alternative for both prevention and treatment of oral mucositis. Its main advantages include being a noninvasive treatment and its low cost.* M. recutita* provides effective results in reducing inflammatory activity, acceleration of the process of repair, and promoting analgesia. However, due to great variability in the various types of intervention, it is necessary that more studies be developed with appropriate sample size and methodology to confirm the best method of intervention in the management of oral mucositis.

## Figures and Tables

**Table 1:** Scale of assessment of the quality of clinical studies.

**Items**
1. The study was described as random (using words such as “random”, “randomization”)?
2. The study was described as double blind?
3. There have been comparisons and results?
4. Comparisons and results described are adequate?
5. Losses have been described and exclusions?

**Table 2:** Articles included in the review, organized in chronological order of publication: authors, year, type of cancer, treatment modality, lesion classification instrument, study groups, number of patients, form of administration, and duration of the intervention.

**Reference **	**Type of cancer**	**Treatment modality**	**Injury classification tool**	**Study groups**	**Number of patients**	**Form of administration**	**Duration of the intervention**
[11]	Head and neck cancer	QT	The WHO scale	Group 1. Chamomile-Prophylaxis (radiotherapy) Group 2. Chamomile Prophylaxis (chemotherapy) Group 3. Therapeutic Chamomile	Group 1: 20 Group 2: 46 Group 3. 32	Mouthwash	14 consecutive days

[12]	NC	QT	The WHO scale	Group 1: Placebo Group 2: Chamomile	Group 1: 82 Group 2: 82	Mouthwash	14 consecutive days

[13]	NC	QT	The WHO scale	Group: Chamomile	Group: 01	Oral	30 consecutive days

[14]	Head and neck cancer	RT	The WHO scale	Group 1. Chamomile gel during the radiotherapy Group 2. Chlorhexidine digluconate Group 3. Chamomile gel after radiotherapy	Group 1: 7 Group 2: 7 Group 3: 8	Topic	NC

[8]	Leukemia	TCH	The WHO scale	Group 1. Control Group 2. the 0.5% Group 2 Chamomile: Chamomile to 1% Group 3. the chamomile 2%	Group 1: 10 Group 2: 10 Group 3: 10 Group 4: 10	Mouthwash	5 consecutive days

[15]	Head and neck cancer	RT	The WHO scale	Group 1. Chamomile Group 2. Honey Group 3. Water	Group 1: 35 Group 2: 35 Group 3: 35	Mouthwash	15 consecutive days

[16]	Leukemia	TCT	The toxicity scale INC	Group 1. Placebo Group 2. Mint and Chamomile	Group 1. 33 Group 2. 27	Mouthwash	NC

[17]	Gastric cancer	QT	The WHO scale	Group 1. Cryotherapy-Control Group 2. Cryotherapy with chamomile	Group 1. 18 Group 2. 20	Topic	22 consecutive days

[18]	Non-Hodgkin lymphoma	RT	Visual analogue scale sterile caliper	Group 1. Placebo Group 2. Triamcinolone Acetonide Group 3. Chamomile	Group 1: 15 Group 2: 14 Group 3: 14	Oral	10 consecutive days

[19]	Acute lymphoblastic leukemia	QT	The WHO scale	Single group: Chamomile	Single group: 31	Mouthwash	14 consecutive days

[20]	Acute lymphoblastic leukemia	QT	The WHO scale	Group 1: (Allopurinol, sucralfate, sodium bicarbonate, and semi-saline serum) Group 2: Chamomile	Group 1: 31 Group 2: 31	Mouthwash	14 consecutive days

UI: uninformed; ↓: reduction; ↑: increase; TNF: tumor necrosis factor; IL: interleukin; HCT: hematopoietic cell transplantation; QT: chemotherapy; RT: radiotherapy; WHO: World Health Organization.

**Table 3:** Clinical trials on the effectiveness of *Matricaria*.

**Reference**	**Intervention**	**Jadad** **score**	**Results**
[11]	Kamillan Liquidum solution made from chamomile flowers	4	↓ in the appearance of oral mucositis in 85% of the patients ↑ immediate relief of painful symptomatology ↑ reepithelialization of the desquamated tissue

[12]	Chamomile concentrate	5	Degree of oral mucositis < in the male gender No significant difference (treated versus placebo groups)

[13]	Infusion with 8 g of dried flowers with 20 ml of water	-	↓ in the oral mucositis grade III on the 13th day 30th day complete absence of mucositis

[14]	Gel with 3% of chamomile extract	3	↓ discomfort and severity of oral mucositis ↓ treatment time It was not effective in preventing

[8]	Oral antiseptic with 0.5%, 1%, and 2% of chamomile extract	5	Significant results found in the dosage of 1% ↓ incidence ↓ intensity of injuries ↓ oral mucositis duration

[15]	3 ml of chamomile mouthwash added to the half cup of water	3	↓ pain ↓ incidence

[16]	Oral antiseptic with 1% of chamomile extract and 1% of	5	↓ pain ↓ dryness of the oral cavity ↓ dysphagia

[17]	Ice with 400 ml of water and 10 g of chamomile flowers	4	↓ incidence of oral mucositis ↓ painful symptomatology ↓ presence of ulcerations

[18]	Chamomile Orobase	5	↓ intensity of pain ↓ the size of the lesion

[19]	15 drops of chamomile solution diluted in a glass of water	0	↓ incidence ↓ gravidity

[20]	30 ml of chamomile	4	↓ incidence ↓ gravidity

↓: reduction; ↑: increase.

**Table 4:** Articles included in the review organized in chronological order of publication: authors, animal experiment, study groups, intervention, duration of the experiment, and results.

**Reference**	**Animal**	**Experiment**	**Study groups**	**Intervention**	**Duration of the experiment**	**Results**
[21]	Albino male rats (Wistar)	Mucosal ulceration left by abrasion with scalpel blade and marker of 8 mm diameter	Group 1. Treated with saline Group 2. Treated with chamomile extract Group 3. Treated with triamcinolone	NC	10 days	Epithelium refurbished absence of inflammation ↑ collagen deposition ↓ apoptosis and TNF-*α*

[22]	Albino male rats (Wistar)	Injury with 1 mm of depth in the central region of the tongue	Group 1. Chamomile ointment Group 2. Without treatment	Topical application of 2 ml chamomile extract with concentration of 10%	10 days	↑ epithelialization and collagenous fibers ↑ fibroblasts ↓ inflammation

[23]	Female albino rats (Wistar)	Intraperitoneal administration of 5-Fluorouracil	Group 1. Distilled water management Group 2. Chamomile extract	Probe intragastric administration of the alcoholic extract of chamomile	12 days	↓ toxicity caused by medicine ↓ size of the lesion prolonged use caused toxicity in the mucosa of the tongue

[24]	Female albino mice (Swiss)	Application of injection of carrageenan on hind right mouse	Group 1. Saline Group 2. Chamomile	MeOH extract of chamomile	16 days	↓ development of arthritis ↓ histamine ↓ inflammation

[25]	Albino male rats (Wistar)	The animals were trichotomized with an electric razor in an area of skin back approximately 10 cm	Group 1. Ethanolic extract of Chamomile Group 2. Crude extract of Chamomile	Topical application in skin of Wistar rats with carrageenan-induced paw edema	12 days	Not provide anti-inflammatory action on the induced edema

[9]	Male Golden Syrian hamsters	Intraperitoneal administration of 5-Fluorouracil	Group 1. Without treatment Group 2. Chamomile Group 3. Corticosteroids	Ointment with 100 g of chamomile extract	14 days	↑ levels of TNF-*α* on the 5th day ↓ severity ↓ levels of IL-1*β* and TNF-*α*

[26]	Female Syrian Golden hamsters	Intraperitoneal administration of 5-Fluorouracil	Group 1. Without treatment Group 2. Chamomile Group 3. Corticosteroids	Ointment with 100 g of chamomile extract	16 days	↓ incidence of oral mucositis ↓ severity ↓ vascular hyperemia ↓ inflammatory infiltration

[27]	Albino male rats (Wistar)	Intraperitoneal administration of 5-Fluorouracil	Group 1. Without treatment Group 2. Chamomile	Ointment with 10% of chamomile extract	10 days	↑ reepithelialization ↑ collagen fibers ↓ of the inflammatory process ↓ on wound size

[28]	Albino male rats (Wistar)	Traumatic ulcers on tongue with 3 mm diameter	Group 1. Without drugs Group 2. Chamomile Group 3. Acetone of triamcinolone topical	Topical formulation of Chamomile	14 days	↑ healing ↑ repair of the epithelium and connective tissue total in 5 days ↓ inflammatory cells

[29]	Albino male rats (Wistar)	Immersion in boiling water for 8 seconds, resulting in burning of 20% of the body area	Group 1. Without treatment Group 2. Application of olive oil Group 3. Chamomile	Chamomile flowers folder added to olive oil	67 days	↑ tissue regeneration ↓ inflammatory infiltration

UI: uninformed; ↓: reduction; ↑: increase; TNF: tumor necrosis factor; IL: interleukin.

**Table 5:** Effects of *Matricaria recutita*.

**Characteristics**	**Effects of *M. recutita***
**Histological**	↑ reepithelialization of the desquamated tissue
	↓ vascular hyperemia
	↑ collagen fibers
	↓ inflammatory infiltration
	↓ inflammatory process

**Clinical**	↑ immediate pain relief
	↓ dryness of the oral cavity
	↓ dysphagia
	↓ wound size
	↓ incidence
	↓ duration
	↓ presence of ulcerations
	↓ discomfort
	↓ severity

## Data Availability

The data from this manuscript refer to a bibliographic review.

## References

[1] Legert K. G., Tsilingaridis G., Remberger M. (2015). The relationship between oral mucositis and levels of pro-inflammatory cytokines in serum and in gingival crevicular fluid in allogeneic stem cell recipients. *Supportive Care in Cancer*.

[2] Ardito F., Giuliani M., Perrone D. (2016). Expression of salivary biomarkers in patients with oral mucositis: Evaluation by SELDI-TOF/MS. *Oral Diseases*.

[3] Figueiredo A. L. P., Lins L., Cattony A. C., Falcão A. F. P. (2013). Laser therapy in oral mucositis control: a meta-analysis. *Revista Da Associacao Medica Brasileira*.

[4] Al-Azri A. R., Gibson R. J., Bowen J. M., Stringer A. M., Keefe D. M., Logan R. M. (2015). Involvement of matrix metalloproteinases (MMP-3 and MMP-9) in the pathogenesis of irinotecan-induced oral mucositis. *Journal of Oral Pathology & Medicine*.

[5] Anand A., Anandi P., Jain N. A. (2016). CD34+ selection and the severity of oropharyngeal mucositis in total body irradiation-based allogeneic stem cell transplantation. *Supportive Care in Cancer*.

[6] Krause C. E., Otieno B. A., Bishop G. W. (2017). Ultrasensitive microfluidic array for serum pro-inflammatory cytokines and C-reactive protein to assess oral mucositis risk in cancer patients. *Analytical and Bioanalytical Chemistry*.

[7] Chen P., Mancini M., Sonis S. T. (2016). A Novel Peptide for Simultaneously Enhanced Treatment of Head and Neck Cancer and Mitigation of Oral Mucositis. *PLoS ONE*.

[8] Braga F. T., Santos A. C., Bueno P. C. (2015). Use of Chamomilla recutita in the Prevention and Treatment of Oral Mucositis in Patients Undergoing Hematopoietic Stem Cell Transplantation. *Cancer Nursing*.

[9] Curra M., Martins M. A. T., Lauxen I. S. (2013). Effect of topical chamomile on immunohistochemical levels of IL-1*β* and TNF-*α* in 5-fluorouracil-induced oral mucositis in hamsters. *Cancer Chemotherapy and Pharmacology*.

[10] Jadad A. R., Moore R. A., Carroll D. (1996). Assessing the quality of reports of randomized clinical trials: Is blinding necessary?. *Controlled Clinical Trials*.

[11] Carl W., Emrich L. S. (1991). Management of oral mucositis during local radiation and systemic chemotherapy: A study of 98 patients. *The Journal of Prosthetic Dentistry*.

[12] Fidler P., Loprinzi C. L., O'Fallon J. R. (1996). Prospective evaluation of a chamomile mouthwash for prevention of 5-FU-induced oral mucositis. *Cancer*.

[13] Mazokopakis E. E., Vrentzos G. E., Papadakis J. A., Babalis D. E., Ganotakis E. S. (2005). Wild chamomile (Matricaria recutita L.) mouthwashes in methotrexate-induced oral mucositis. *Phytomedicine*.

[14] Sampaio T. P., Holmes T. S., De Medeiros Nóbrega D. R. (2014). Evaluation of the effectiveness of *Matricaria recutita*Linn. in the prevention and control of radiation-induced oral mucositis. *Revista Odonto Ciência*.

[15] Bahramnezhad F., Nayeri N. D., Bassampour S. S., Khajeh M., Asgari P. (2015). Honey and radiation-induced stomatitis in patients with head and neck cancer. *Iranian Red Crescent Medical Journal*.

[16] Tavakoli Ardakani M., Ghassemi S., Mehdizadeh M. (2016). Evaluating the effect of Matricaria recutita and Mentha piperita herbal mouthwash on management of oral mucositis in patients undergoing hematopoietic stem cell transplantation: A randomized, double blind, placebo controlled clinical trial. *Complementary Therapies in Medicine*.

[17] dos Reis P. E. D., Ciol M. A., de Melo N. S., Figueiredo P. T. D. S., Leite A. F., Manzi N. D. M. (2016). Chamomile infusion cryotherapy to prevent oral mucositis induced by chemotherapy: a pilot study. *Supportive Care in Cancer*.

[18] Andishe Tadbir A., Pourshahidi S., Ebrahimi H., Hajipour Z., Memarzade M. R., Shirazian S. (2015). The effect of Matricaria chamomilla (chamomile) extract in Orabase on minor aphthous stomatitis, a randomized clinical trial. *Journal of Herbal Medicine*.

[19] Pourdeghatkar F., Motaghi M., Darbandi B., Baghersalimi A. (2017). Comparative effect of chamomile mouthwash and topical mouth rinse in prevention of chemotherapy-induced oral mucositis in Iranian pediatric patients with acute lymphoblastic leukemia. *Iranian Journal of Blood and Cancer*.

[20] Pourdeghatkar F., Motaghi M., Darbandi B., BagherSalimi A. (2017). The Effect of Chamomile Mouthwash on the Prevention of Oral Mucositis Caused by Chemotherapy in Children with Acute Lymphoblastic Leukemia. *Iranian Journal of Pediatric Hematology and Oncology*.

[21] Oliveira B. V., De Barros Silva P. G., Nojosa J. D. S. (2016). Tnf-alpha expression, evaluation of collagen, and TUNEL of matricaria recutita L. extract and triamcinolone on oral ulcer in diabetic rats. *Journal of Applied Oral Science*.

[22] Nayak B. S., Raju S. S., Rao A. V. C. (2007). Wound healing activity of Matricaria recutita L. extract.. *Journal of Wound Care*.

[23] AlRefai A. S. (2014). Effect of Chamomile Extract on the Tongue of Chemotherapy Treated Albino Rats (Histopathological and Immunohistochemical Study). *Journal of Clinical & Cellular Immunology*.

[24] Katti H., Singh P., Ramkishan A., Chandrashekhar V., Sowmya C., Panji M. (2014). Anti-inflammatory Activity of Matricaria recutita L. against Acute and Chronic Inflammatory Models. *Science, Technology and Arts Research Journal*.

[25] Queiroz M. B. R., Lucena G. R. S., Caldas E. D., Silva M. V. (2014). Evaluation of the anti-inflammatory activity of gel with Matricaria recutita L. using a permeation enhancer. *Rev Bras Farm*.

[26] Pavesi V. C. S., Lopez T. C. C., Martins M. A. T. (2011). Healing action of topical chamomile on 5-fluouracil induced oral mucositis in hamster. *Supportive Care in Cancer*.

[27] Duarte C.-M., Quirino M.-R., Patrocínio M.-C., Anbinder A.-L. (2011). Effects of Chamomilla recutita(L.) on oral wound healing in rats. *Medicina Oral Patología Oral y Cirugía Bucal*.

[28] Martins M. D., Marques M. M., Bussadori S. K. (2009). Comparative analysis between *Chamomilla recutita* and corticosteroids on wound healing. An *in vitro* and *in vivo* study. *Phytotherapy Research*.

[29] Jarrahi M. (2008). An experimental study of the effects of *Matricaria chamomilla* extract on cutaneous burn wound healing in albino rats.. *Natural Product Research (Formerly Natural Product Letters)*.

[30] Ferreira E. B., Vasques C. I., Jesus C. A. C., Reis P. E. D. (2015). Topical effects of Chamomilla Recutita in skin damage: A literature review. *Pharmacologyonline*.

[31] Seyyedi S.-A., Sanatkhani M., Pakfetrat A., Olyaee P. (2014). The therapeutic effects of chamomilla tincture mouthwash on oral aphthae: A randomized clinical trial. *Journal of Clinical and Experimental Dentistry*.

[32] Macedo R. A., Morais E. F., Dantas A. N., Morais M. d. (2015). Chlorhexidine to treat oral mucositis in patients with acute leukemia: systematic review. *Revista DOR *.

[33] Ramos-e-Silva M., Ferreira A. F., Bibas R., Carneiro S. (2006). Clinical evaluation of fluid extract of Chamomilla recutita for oral aphthae.. *Journal of drugs in dermatology : JDD*.

[34] Bhaskaran N., Shukla S., Srivastava J. K., Gupta S. (2010). Chamomile: an anti-inflammatory agent inhibits inducible nitric oxide synthase expression by blocking RelA/p65 activity. *International Journal of Molecular Medicine*.

[35] Gomes R. N. S., LAGO E. C. (2016). Oral health care in Brazil: current panorama. *Revista Facema*.

